# Hemichorea-Hemiballism Syndrome in a Patient With Diabetic Striatopathy and Lacunar Stroke

**DOI:** 10.7759/cureus.94238

**Published:** 2025-10-09

**Authors:** Izabel Antova, Nikolay Y Yordanov, Dimitar Taskov, Nikolay Topalov, Milena Milanova

**Affiliations:** 1 Department of Neurology, Multiprofile Hospital for Active Treatment in Neurology and Psychiatry "St. Naum", Sofia, BGR; 2 Department of Radiology, Multiprofile Hospital for Active Treatment in Neurology and Psychiatry "St. Naum", Sofia, BGR

**Keywords:** diabetic striatopathy, hemichorea-hemiballism, hyperkinetic movement disorder, ischemic stroke, treatment

## Abstract

Hemichorea-hemiballism syndrome is a hyperkinetic movement disorder that is usually associated with stroke within the basal ganglia, thalamus, or subthalamic area. However, it can also occur as a rare manifestation of severe hyperglycemia. The simultaneous or subsequent occurrence of ischemic stroke and diabetic striatopathy has only been reported in a few cases. We present a rare case of acute onset of involuntary movements in the right upper and lower extremities, which was initially thought to be associated solely with hyperglycemia. A non-contrast computed tomography (NCCT) scan of the brain revealed a hyperdense left caudate nucleus and lentiform nucleus. The patient was treated with antidiabetic medications. However, due to the persistence of neurological symptoms, a follow-up CT was performed, which showed evidence of a lacunar stroke in the left caudate nucleus. Treatment with aspirin and haloperidol was administered. On further follow-up, the patient's clinical condition has fully recovered. This case report highlights the potential presence of two independent conditions that can manifest as an identical neurological condition. Prompt and accurate identification of those conditions is crucial for avoiding misdiagnosis and delays in treatment.

## Introduction

Hemichorea-hemiballism (HC-HB) is a rare hyperkinetic neurologic disorder characterized by unilateral or bilateral involuntary, jerky movements involving the face, trunk, upper and/or lower extremities. Due to their non-rhythmic, continuous, and irregular nature, such movements resemble flinging [1,2]. Ischemic (IS) or hemorrhagic stroke within the basal ganglia, thalamus, or subthalamic area is the most common cause of this clinical presentation. Another condition presenting with HC-HB is diabetic striatopathy (DS) - a rare manifestation of severe hyperglycemia, predominantly documented in patients with type 2 diabetes mellitus (DM) [3]. Structural lesions, including vascular malformations or demyelinating lesions, and infectious disorders, such as CNS toxoplasmosis and tuberculoma, can also cause this neurological condition [2,4].

We present a case of a patient with DS, with concurrent lacunar stroke in the caudate nucleus detected on follow-up imaging. Given the scarcity of reported cases of hyperglycemia-induced striatopathy and the limited data available on the development of subsequent or concurrent lacunar stroke in such patients, we believe that our research will contribute valuable insights into this clinical entity. Additionally, we conducted a non-systematic literature review to complement our findings and summarize the characteristics of patient presentations and management, equipping clinicians with essential knowledge for accurately diagnosing and treating patients with HC-HB syndrome.

## Case presentation

A 63-year-old man presented with a three-day history of continuous and involuntary movements in his right upper and lower extremities. His medical history included an orchiectomy at the age of 45 due to testicular cancer, for which there is currently no evidence of relapse. The patient reported no history of diabetes mellitus or chorea. He was not on any medications.

Upon examination, his blood pressure measured 140/80 mmHg, with a pulse of 78 beats per minute. His oxygen saturation (SpO_2_) was 98% on room air, and his lungs were clear upon auscultation. Notably, the physical examination revealed high-amplitude involuntary dyskinesias in the right extremities. Athetotic and torsional movements were observed in the right shoulder, elbow, wrist, hip, and ankle (Video 1).

**Video 1:** Initial presentation

**Figure 1:**
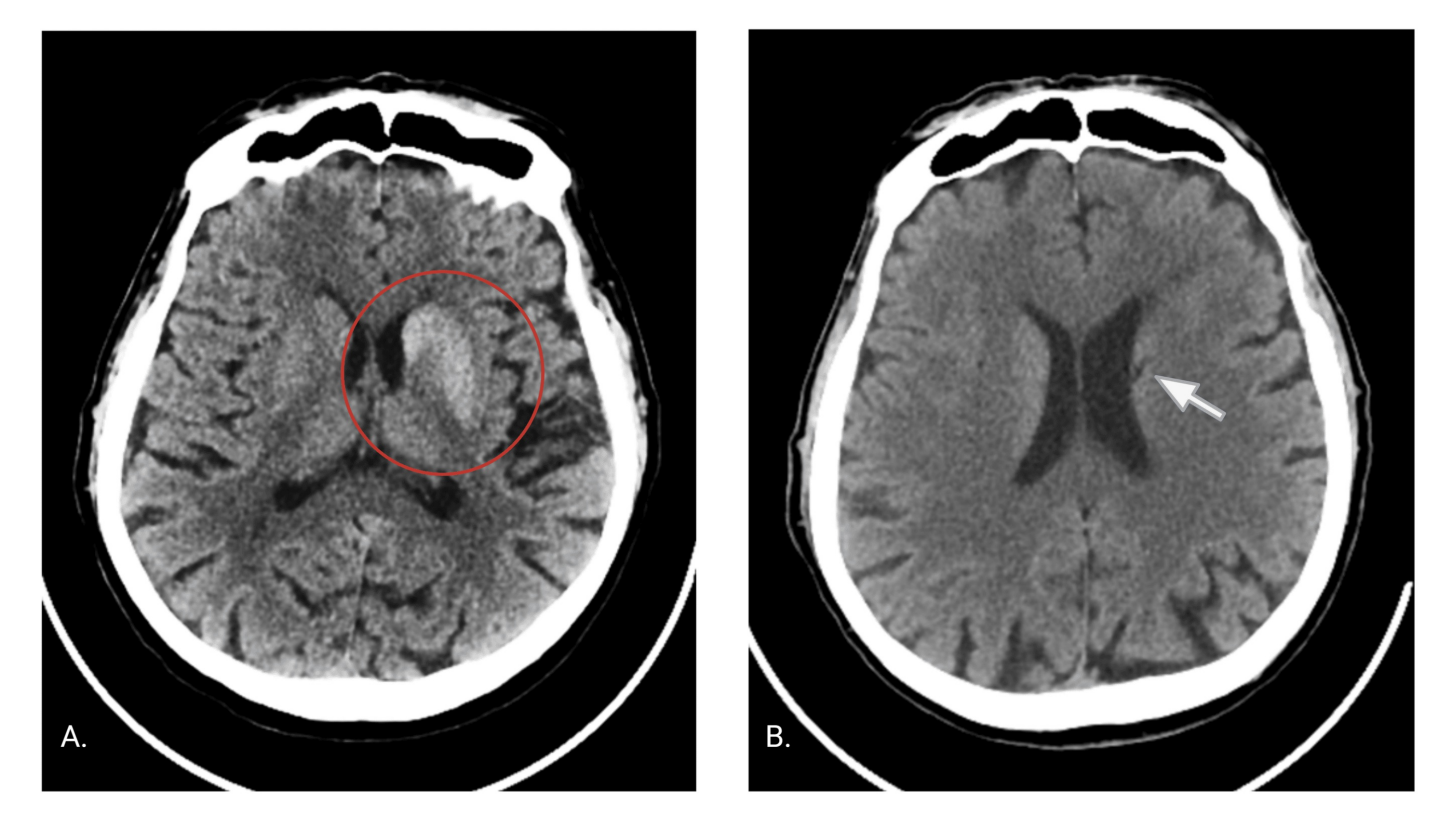
Patient's non-contrast computed tomography (NCCT) of the brain (A) Initial non-contrast CT scan. Marked hyperdensity within the caudate nucleus and the putamen can be observed. (B) Non-contrast CT scan obtained on follow-up. Marked hypodensity within the tail of the caudate nucleus, corresponding to a lacunar stroke.

During the gait examination, the patient presented with jerky movements in his right foot, resulting in an irregular and unsteady gait. Coordination tests, which included right finger-to-nose and heel-to-shin movements, revealed sinuous motions due to choreoathetosis; however, the patient was able to reach the targets. Additionally, he exhibited brisk deep tendon reflexes on the right side. The cranial nerves, sensory examination, and other systemic assessments were unremarkable. Laboratory tests indicated a random glucose level of 30.63 mmol/L (551.3 mg/dL) and glycosuria without ketonuria. The patient's glycated hemoglobin (HbA1c) was 28.1%. Full blood count, liver function tests, and renal function tests all returned normal results. Non-contrast computed tomography (NCCT) of the brain showed a hyperdense left caudate and lentiform nuclei (Figure 1A).

Given the absence of a family history of chorea, the characteristic NCCT brain findings, and the results from the blood samples, a tentative diagnosis of DS was made. Initial management included intravenous administration of a rapid-acting insulin analog under tight glycemic control. Following stabilization of glycemic levels, oral antihyperglycemic therapy with metformin was initiated, subsequently supplemented with a sulfonylurea agent.

Three months later, the patient showed substantial improvement in his movement disorder; however, subtle jerky movements were evident (Video 2).

**Video 2:** First follow-up

His blood sugar level was 5.9 mmol/L (106.2 mg/dL), and his HbA1C was 5.73%. The NCCT showed no hyperdensity in the basal ganglia, but a lacunar stroke in his left caudate nucleus was detected (Figure 1B).

The patient was treated with aspirin and haloperidol and was discharged after five days of admission in a better condition with an improvement of his HC-HB symptoms. On further follow-up, the patient fully recovered (Video 3).

**Video 3:** Second follow-up

## Discussion

In addition to the clinical case presented, a non-systematic literature review was performed to provide a comprehensive analysis of the causes, clinical presentation, associated disorders, and management of HC-HB and to provide more context into why our case is extremely rare in terms of etiology and disease progression. The MEDLINE database was searched, with the following terms used: "hyperdense basal ganglia" and "non-ketotic hyperglycemia". Articles describing clinical cases of diabetes-induced striatopathy, written in English, with available full text, were selected. Additional research items that were relevant to the clinical case, but were discovered from reference lists, were also taken into consideration. As a second step in our strategy, case reports, including a constellation of IS stroke or lacunar stroke and diabetic striatopathy, were reviewed to specifically identify patients with a similar etiological constellation as the presented case.

A total of 65 patients with a diagnosis of diabetic striatopathy were identified in our search - 46 female and 19 male patients bearing this diagnosis (female-to-male ratio accounting for 2.4) [5-23]. The mean age of the identified patients was 62. There were only six reported cases with concomitant diabetes-induced HC-HB syndrome and acute IS stroke involving the basal ganglia [24-29].

Etiology and differential diagnosis

Although HC-HB commonly occurs due to IS or hemorrhagic stroke within the posterolateral putamen, which is supplied by the lenticulostriate arteries, it is a consequence of less than 1% of all strokes. Hyperkinetic movements can occur immediately after the onset of acute stroke or be of delayed onset [4]. Disruption of the dopamine regulation is suggested to be the leading mechanism by which stroke can elicit this condition [29]. While the subthalamic nucleus has been considered a classical area related to hemichorea, lesions affecting other regions such as the caudate, putamen, cortex, thalamus, and globus pallidus have also been found to induce this state [4,30]. The functional outcome is better in patients with cortical lesions [30]. Management of this condition usually includes treatment with dopamine antagonists [4].

Diabetic striatopathy is a complication in patients with diabetes mellitus and can present with HC-HB. The exact pathophysiology of this condition is not fully understood, but several possible explanations exist. These include hyperglycemia-induced disruption of the blood-brain barrier, which can lead to local metabolic derangements, and decreased GABAergic transmission in the striatum, which may occur due to the enhanced utilization of GABA as an alternative energy source in states of hyperglycemia-induced anaerobic metabolism [31,32].

In sporadic cases, diabetic striatopathy together with concurrent IS stroke can be the cause of the HC-HB clinical syndrome. According to the literature review that was carried out, only six cases of such a scenario have been described so far [24-29]. In our case, chronic microvascular disturbance in the striatum may be the common pathological mechanism for IS and DS. The IS lesion is supposed to be induced by hyperglycemia because of plasma osmolarity and hyperviscosity [33].

Clinical features

The most commonly reported signs and symptoms in DS include acute-onset, involuntary, dance-like movements affecting the face and upper and/or lower limbs (Table 1) [5-23], although tremors, seizures, and focal dystonia have been increasingly reported as presenting motor signs [34].

**Table 1:** Body involvement of patients diagnosed with diabetic striatopathy UP – upper limb, LL – lower limb Numbers indicate the number of patients reported in the reviewed literature with the respective clinical involvement.

Part of body involved	Unilateral (n=38)	Bilateral (n=4)
UL or LL only	16	1
UL or LL + facial involvement	2	1
UL & LL	17	0
UL & LL + facial involvement	3	2

Less commonly reported presenting symptoms of patients with radiologically confirmed diabetic striatopathy include, but are not limited to, memory loss, increased length and frequency of sleep, loss of consciousness, progressively worsening generalized weakness and lightheadedness, and stroke-mimicking symptoms such as weakness, slurred speech, and facial droop [12,13,15,19].

The mean blood glucose level within our reviews is 29.3 mmol/L (range: 12.13-55.3), SD = 7.9, and the mean for the reported HbA1c is 13.9%, SD = 3.9.

Involved brain structures and imaging findings

The diagnosis of diabetic striatopathy is confirmed by medical imaging. This condition is characterized by hyperdensity on CT and hyperintensity on T1-weighted MRI in the striatum, on the side that is contralateral to the symptoms [6]. The imaging presentation of DS is usually reversible, with long-term uncontrolled hyperglycemia being a major cause of irreversible damage. The most commonly affected structure on imaging is the putamen. Combined involvement of the putamen and caudate nuclei also occurs, albeit at a lower rate. Rarely, all structures comprising the striatum can be affected [35]. There are no reported cases of isolated involvement of the caudate or the lentiform nuclei. The affected parts of the brain exhibit increased attenuation on CT, hyperintensity on T1-weighted MRI, and hypo-, hyper-, or isointensity on T2-weighted MRI [36].

Out of the 65 patients identified in our literature review, an unremarkable CT scan was found in four patients [9,10,12], and an unremarkable T1WI was reported in one patient [10]. The imaging findings in the patients reported in the articles included in the small-scale literature review are depicted in Table 2.

**Table 2:** Imaging findings in patients diagnosed with diabetic striatopathy MRI – magnetic resonance imaging; NCCT – non-contrast computed tomography Numbers indicate the number of patients reported in the reviewed cases presenting with the corresponding imaging characteristics.

Brain structure involvement	T1-weighed MRI	T2-weighed MRI			NCCT
	Hyper-intensity	Hyper-intensity	Hypo-intensity	Iso- intensity	Hyper-density
Unilateral involvement of					
Only the nucleus caudatus	0	0	0	0	1
Only the globus pallidus	1	1	0	0	2
Only putamen	6	0	8	0	6
Nucleus caudatus + putamen	5	0	1	0	3
Globus pallidus + putamen	1	0	0	0	0
Nucleus caudatus + globus pallidus + putamen	5	8	7	0	7
Right paracentral midbrain + anterior aspect of the right cerebral peduncle	1	0	0	0	0
Bilateral involvement of					
Only the nucleus caudatus	0	0	0	0	2
Only the globus pallidus	1	1	1	0	1
Nucleus caudatus + globus pallidus	1	0	0	0	0
Nucleus caudatus + globus pallidus + putamen	1	0	0	2	0

Outcomes: In all the cases that were reviewed, a significant improvement in clinical symptoms and motor function upon normalization of blood glucose levels was reported within the timeframe of patients’ hospital stay. In only nine articles, data from follow-up examinations were reported [7,9,11,12,14,16,20,22,23]. Due to the non-standardized follow-up strategy and the differences in reported data among the reviewed articles, concrete conclusions cannot be made. However, full resolution was reported in eight of those nine cases, and in one patient, the symptoms were still present at follow-up, albeit less severe [7]. In conclusion, less severe symptoms were present in patients with optimized blood glucose levels.

Lancellotti et al. presented the first case in the literature of a patient with the simultaneous occurrence of diabetes-induced HC-HB syndrome and IS stroke. The patient experienced three stroke recurrences and was symptom-free only after successful glycemic control [27]. Carrion et al. described a patient who developed a stroke two weeks after presenting with diabetic striatopathy and highlighted that poor glycemic control increases the risk of cerebrovascular events [28]. Lin et al. reported a patient who had undergone two episodes of hemichorea symptoms, with evidence of both hyperglycemia and radiological confirmation of basal ganglia lacunar infarct, and recovered after optimization of blood glucose level [29].

The results from the literature, as well as the data from the presented patient, confirm the observation of Lin et al. that, in patients with dyskinesia symptoms caused by non-ketotic hyperosmolar hyperglycemia, the symptoms substantially improve upon achieving glycemic control [22]. We can also conclude that DM potentiates cerebrovascular events, and their co-occurrence takes a higher toll on patients diagnosed with diabetic striatopathy.

## Conclusions

In rare cases, diabetic striatopathy and ischemic stroke, both of which can cause HC-HB syndrome, may occur simultaneously or subsequently. Practicing endocrinologists, neurologists, and general practitioners should be acquainted with the aforementioned causes of HC-HB so that misdiagnosis, delays in treatment, and incorrect conclusions about the prognosis can be avoided. The diagnosis is confirmed by clinical findings and history, imaging of the brain (CT or MRI), and laboratory values of glucose and/or HbA1. Prognosis is favorable in most cases in which appropriate blood glucose control has been established. The concurrent presence of ischemic lesions in the basal ganglia requires additional treatment and worsens recovery and outcome.
